# Editorial: Clinical aspects of different forms of diabetes in children and adolescents

**DOI:** 10.3389/fendo.2022.1110373

**Published:** 2022-12-19

**Authors:** Stefano Zucchini

**Affiliations:** Istituto di Ricovero e Cura a Carattere Scientifico (IRCCS) Azienda Ospedaliero-Universitaria di Bologna, Bologna, Italy

**Keywords:** type 2 diabetes, type 1 diabetes, monogenic diabetes, MODY, diabetes complications, diabetes prevention

Diabetes is a complex and heterogeneous disease characterized by chronic hyperglycemia causing progressive multiorgan damages in the body, i.e. typical diabetes complications. Although insulin is the most used drug to prevent elevated glucose levels, it is equally necessary to postpone complications by fighting both hypertension and dyslipidemia. Traditionally, diabetes is classified into type 1 diabetes (T1D), type 2 diabetes (T2D) and other specific types; T1D is associated with autoimmune β-cell destruction and mainly starts in childhood, whereas T2D is associated with obesity, insulin resistance and inability of β-cell to compensate for the increased requirements; among other types of diabetes, we can list monogenic diabetes, diseases of the exocrine pancreas and drug- or chemical-induced diabetes. Less frequent forms of diabetes are associated with rare diseases and syndromic pictures (1).

T1D and T2D are heterogeneous diseases whose clinical presentation and progression may vary considerably. Despite classification being important to determine therapy, some individuals cannot be clearly labeled as having T1D or T2D at the time of diagnosis. Similarly, the minority of patients classified as having T1D b (2), i.e. autoantibody negative, are challenging, since it is often difficult to establish whether they are affected by a classic T1D, or, conversely, by a disease in which the destruction of the β-cells is the outcome of either a specific viral aggression, or a genetic form of diabetes.

As a matter of fact, individuals with maturity-onset diabetes of the young [MODY] are often misdiagnosed as having T1D (3); furthermore, autoantibody-negative ‘type 1’ patients of African or Asian origin may present episodic DKA, showing varying degrees of insulin deficiency between episodes (4). Therefore, the clinical presentation is not always helpful in identifying the subtype of diabetes the patient is affected by.

The present issue contains eight interesting manuscripts dealing with T1D prevention, diabetes physiopathology and diagnostic problems and finally with the risk factors of complications, i.e. hyperglycemia and dyslipidemia.

As for T1D prevention, the Finnish manuscript including the case series by the Diabetes Prediction and Prevention birth cohort study *(* Helminen et al. *)* describes early glucose metabolism abnormalities in the progressor vs non progressor children. The various studies led in this country on this cohort have significantly improved the knowledge of the pre-T1D phases leading to overt diabetes. The study contained in this issue led in more than a thousand children, identified dysglycemia and increasing HbA1c values before the presentation of clinical T1D as the hallmark of imminent diabetes onset. This is important, above all, in light of future trials aimed at postponing or preventing the development of T1D.

Moving to diagnostic issues, the study by the Italian pediatricians from Genoa *(* Lezzi et al. *)* specifically addresses the aforementioned issue of the misdiagnosis of MODY cases. By examining with a 28 panel NGS analysis the 23 patients without autoimmunity, they found that 10% were affected by monogenic diabetes: they identified two novel genetic variants, affecting either the NEUROD1 gene or the INS gene. The latter showed DKA at onset, bringing to light once again the difficulties that may arise when distinguishing between the various forms of diabetes simply on the basis of the clinical picture. The study allowes broadening the spectrum of mutations that cause monogenic diabetes in children. It is important that adult diabetologists become aware too that a significant proportion of lean adults diagnosed as T2D may instead have a monogenic congenital form of diabetes.

The study by Cao et al. described a unique case of a young man presenting a series of conditions: muscular dystrophy, an early onset (19 years of age) of autoantibody-negative diabetes and complex phenotype abnormalities, including non-endocrinological and endocrinological diseases, associated with a large deletion (haploid deficiency of 94 genes) within chromosome 8p. Even though some neurological diseases are associated with diabetes, the case presented is a unique mixture of different pathological conditions that are dealt with multiple disciplines. The authors hypothesized that diabetes was caused by complex interactions of known and unknown genetic and environmental factors.

To underline the complexity of glucose metabolism and its relationship with other hormones, Xu et al. examined glucose tolerance *via* OGTT in a large series of patients affected by a rare form a congenital adrenal hyperplasia (17-hydroxylase/17,20-lyase deficiency), which is characterized by chronic hypokaliemia and high progesterone levels. They found that more than 10% of lean patients examined showed glucose metabolism abnormalities up to diabetes, with a direct relationship with hypokalemia and elevated progesterone levels. The latter it is known to cause insulin resistance, representing, in fact, a contributing factor of gestational diabetes.

Hypokalemia is also mentioned in the study by Ge et al. who described for the first time a patient with MODY 5 with diabetes onset in DKA. MODY5 is a rare disease characterized by multiple kidney cysts, pancreatic dysplasia, hypomagnesemia, and diabetes in young adults, usually caused by mutations in HNF1B. The 26 year old Chinese patient showed potassium level of 3.3 mEq/L that may have contributed to the initial ketosis.

The Italian narrative review by the pediatric group of Piccolo et al. again from Genoa encompasses the possible influence on T1D onset in children and the need of specific vaccination of the principal infectious diseases, starting from tuberculosis, pneumococcus and influenza, Covid-19 (under study as a possible trigger for T1D development), gastrointestinal, fungal and dermatological infections and others. The debate is on how infectious diseases may cause or accelerate T1D onset and, at the same time, how they can decompensate an already existing disease, leading to DKA.

Finally, two studies deal with diabetes complications, the first led in children the second in adult patients. The pediatric study by Colinet and Lysy evaluates the effects of posthypoglycemic hyperglycemia (PHH) in children and adolescents with T1D. PHH was found in 22% of the whole cohort, despite showing a different pattern in children and adolescents. The phenomenon is well known in children and adolescents with T1D, since they often overcorrect the episodes of hypoglycemia with an excessive intake of carbohydrates. The study included in this issue has the merit to examine in details the variables influencing PHH; it shows that young and lean children are more prone to experience a short time hypoglycemia, that recovers with hyperglycemia, whereas adolescents and obese children tend to experience hyperglycemia of longer duration. On the other hand, the fear of hypoglycemia is a variable that knowingly affects both parents and patients, preventing the attainment of a good metabolic control. It would be interesting to design a study that simultaneously examined both aspects of the problem, i.e. PHH and fear of hypoglycemia.

The Jordan study led in adult patients with T2D *(* Hyassat et al. *)* focuses on dyslipidemia. The authors found that unfortunately 95% of the patients showed dyslipidemia, with the most common association being high triglycerides-low HDL-c. In addition, more than 90% of patients were overweight or obese subjects and some of them were smokers and/or hypertensive. The paper underlines the difficulties in managing patients with T2D, that are increasingly becoming a worldwide burden for the National Health Services of most countries and a frequent cause of premature death. Furthermore, the age at T2D onset is progressively decreasing, with adolescents not rarely involved (5).

In conclusion, the manuscripts published in the present issue include many aspects related to glucose metabolism, diabetes development and diagnosis, also thanks to the use of genetic testing. T1D is still a serious disease, which will be hopefully prevented or delayed soon. On the contrary, T2D is a worldwide pandemic and every effort should be addressed towards the attainment of a healthy lifestyle in order to prevent obesity and its complications.

## Author contributions

The author confirms being the sole contributor of this work and has approved it for publication.
